# A First Case of Retroperitoneal Cavernous Hemangioma Resection With Senhance Digital Laparoscopy System

**DOI:** 10.1111/ases.70210

**Published:** 2025-12-14

**Authors:** Yoshiki Murase, Masayasu Aikawa, Yuichiro Watanabe, Taku Honma, Takuya Oba, Yumiko Kageyama, Kenichiro Takase, Yukihiro Watanabe, Hiroaki Ono, Katsuya Okada, Yasumitsu Hirano, Kojun Okamoto, Isamu Koyama

**Affiliations:** ^1^ Department of Gastroenterological Surgery Saitama Medical University International Medical Center Saitama Japan; ^2^ Department of Pathology Saitama Medical University International Medical Center Saitama Japan

**Keywords:** cavernous hemangioma, retroperitoneal tumor, Senhance

## Abstract

Senhance digital laparoscopy system (SDLS) is a surgical support robot featuring eye tracking control, haptic feedback, reusable instruments, and an open cockpit that has gradually gained widespread adoption in gastrointestinal surgery. However, there are no reports on the use of SDLS for retroperitoneal tumors. We present the first case of a retroperitoneal cavernous hemangioma that was successfully treated with the use of SDLS. A 61‐year‐old woman was referred to our hospital due to the detection of a heterogeneous 31 × 26 mm mass in the retroperitoneal area. The preoperative examination failed to confirm the diagnosis; therefore, surgical resection was performed using SDLS for definitive diagnosis and treatment. The tumor was located on the left renal vein and successfully resected without intraoperative complications. The operative time was 185 min, and the pathological examination confirmed the diagnosis of cavernous hemangioma. The SDLS enabled the safe and reliable resection of a retroperitoneal tumor.

## 1 Introduction

The retroperitoneal space is located posterior to the peritoneum and contains the kidneys, associated muscles, and major blood vessels such as the aorta and inferior vena cava [1]. Most retroperitoneal tumors are malignant, with benign tumors accounting for approximately 10% of cases [2]. Among benign lesions found in the retroperitoneum, hemangiomas are uncommon [3]. Senhance digital laparoscopy system (SDLS) (Asensus Surgical US Inc., Durham, NC, USA) is a surgical support robot featuring eye tracking control, haptic feedback, reusable instruments, and an open cockpit [4, 5]. In Europe and the United States, surgeries supported by the SDLS have been performed in several facilities, with surgical outcomes for safety and efficacy [6, 7]. In Japan, SDLS surgery receives the same insurance coverage as laparoscopic surgery. However, there have been no previous reports on the use of SDLS for retroperitoneal tumor resection. Herein, we present the first report in which a cavernous hemangioma originating from the retroperitoneal area was successfully treated with SDLS.

## 2 Case Presentation

**FIGURE 1:**
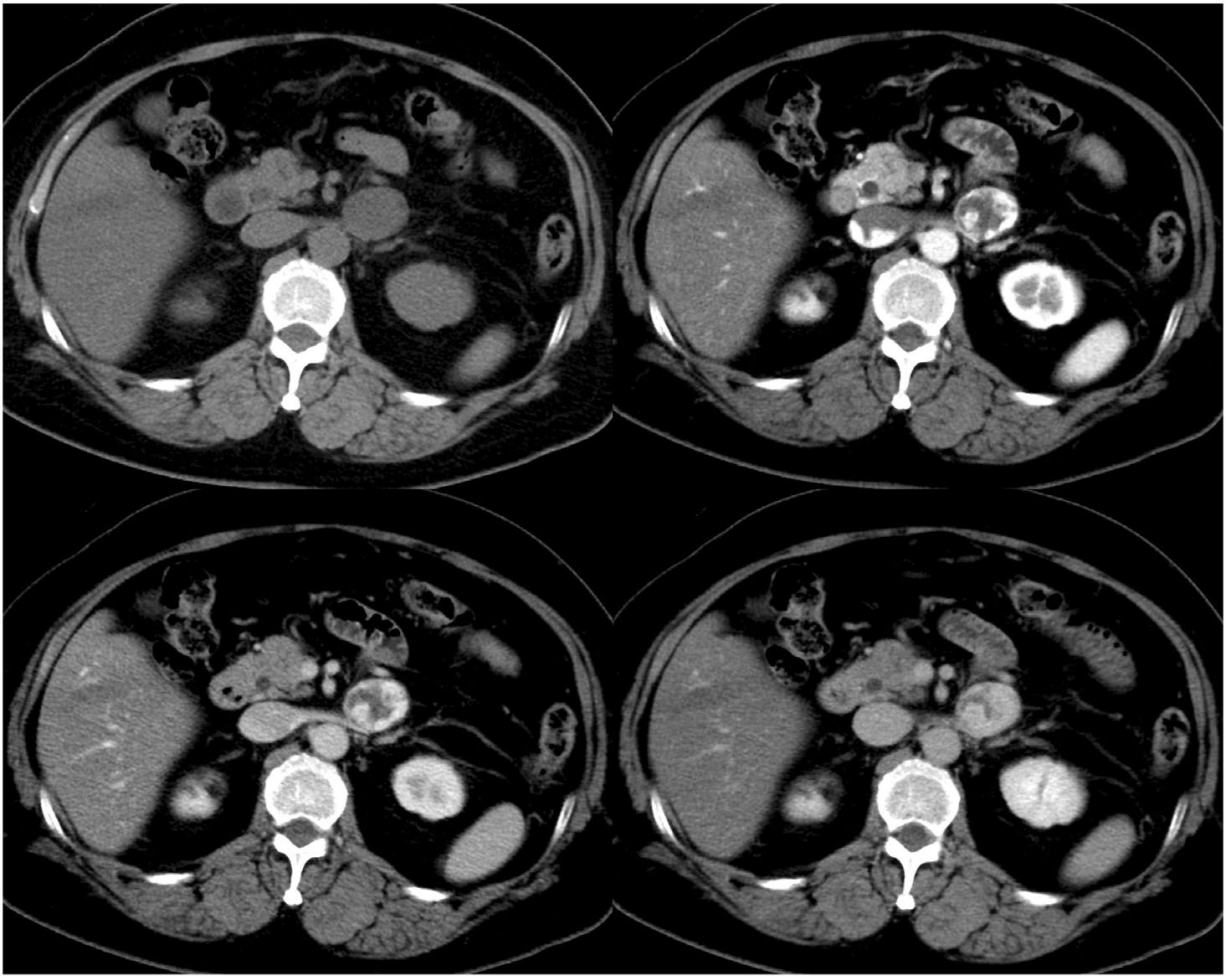
Abdominal CT imaging at the initial diagnosis. CT imaging reveals a 31 × 26 mm gradually heterogeneous enhanced tumor in the retroperitoneal area that does not invade the surrounding tissue.

A 61‐year‐old woman was referred to our hospital from another medical center due to the detection of a retroperitoneal mass. She had a history of hypertension and dyslipidemia. Laboratory results did not indicate elevated concentrations of the tumor markers CA125 (12.1 U/mL), CEA (1.8 ng/mL), or CA19‐9 (6.1 U/mL). Computed tomography (CT) showed a heterogeneous 31 × 26 mm mass with a gradual enhancement in the retroperitoneal space. The tumor was located near the left adrenal gland, but there was no invasion into the surrounding tissue (Figure 1). Positron emission tomography (PET)‐CT, metaiodobenzyl guanidine (MIBG) scintigraphy, and somatostatin receptor scintigraphy demonstrated no increased uptake in the retroperitoneal tumor. An endoscopic ultrasonography‐guided fine‐needle aspiration (EUS‐FNA) of the retroperitoneal tumor was successfully performed, but the diagnosis was not able to be confirmed. Based on the tumor location and the results of preoperative examinations, the tumor was suspected to be a neurogenic tumor. Therefore, for the purposes of diagnosis and treatment, the decision to surgically remove the tumor was made.

**FIGURE 2:**
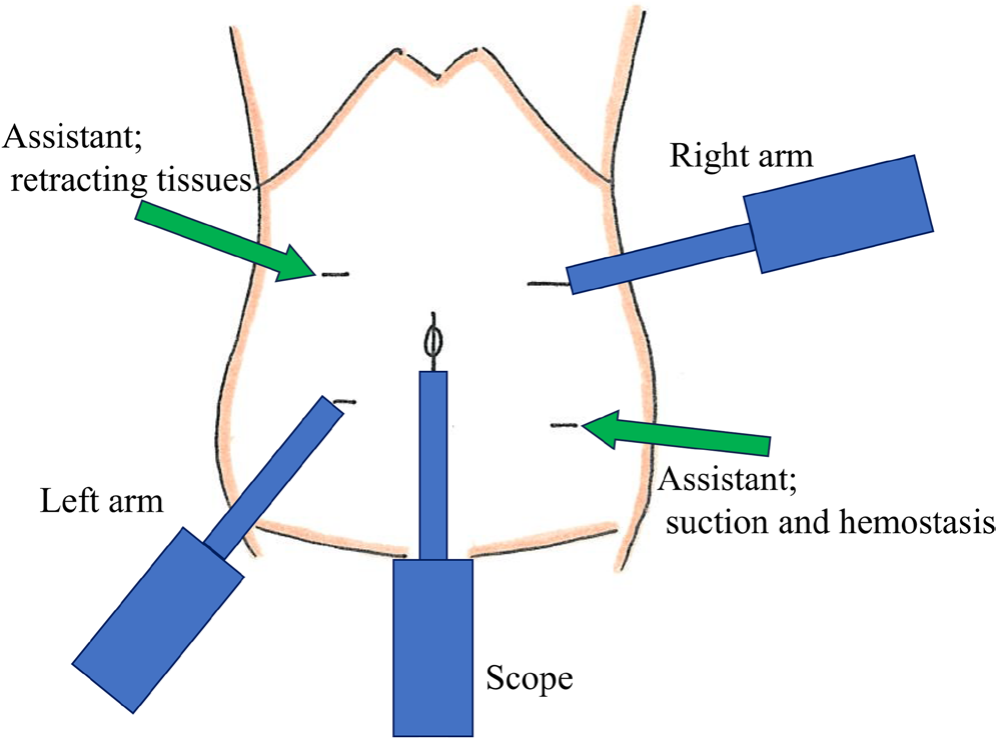
The robot arm placement of this case.

**FIGURE 3:**
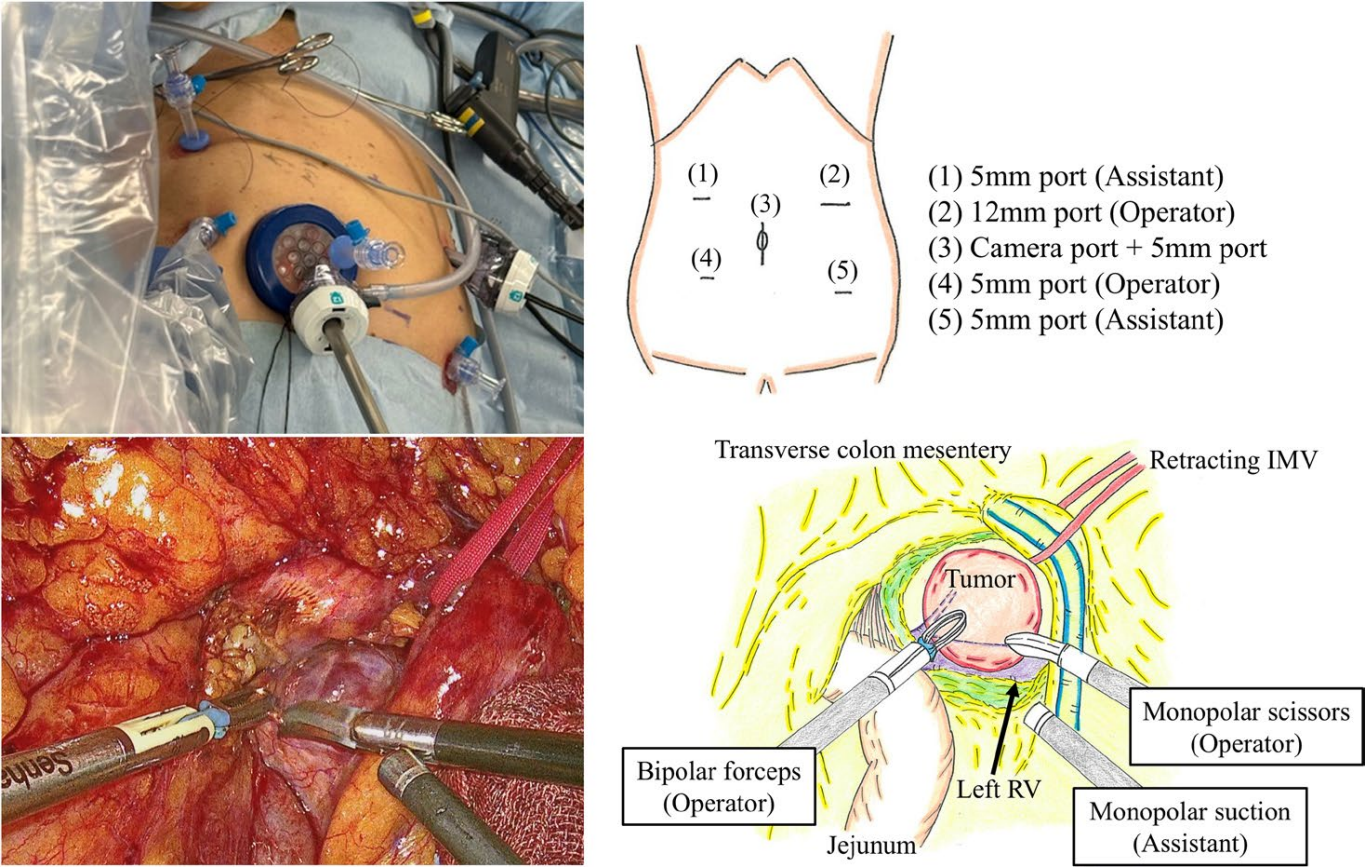
Intraoperative findings and schematic illustrations. The picture and schematic illustration of port placement show relative positions of the ports and the docking arms. Intraoperative findings show the tumor has no invasion surrounding organ or vessels. IMV, inferior mesenteric vein; RV, renal vein.

**FIGURE 4:**
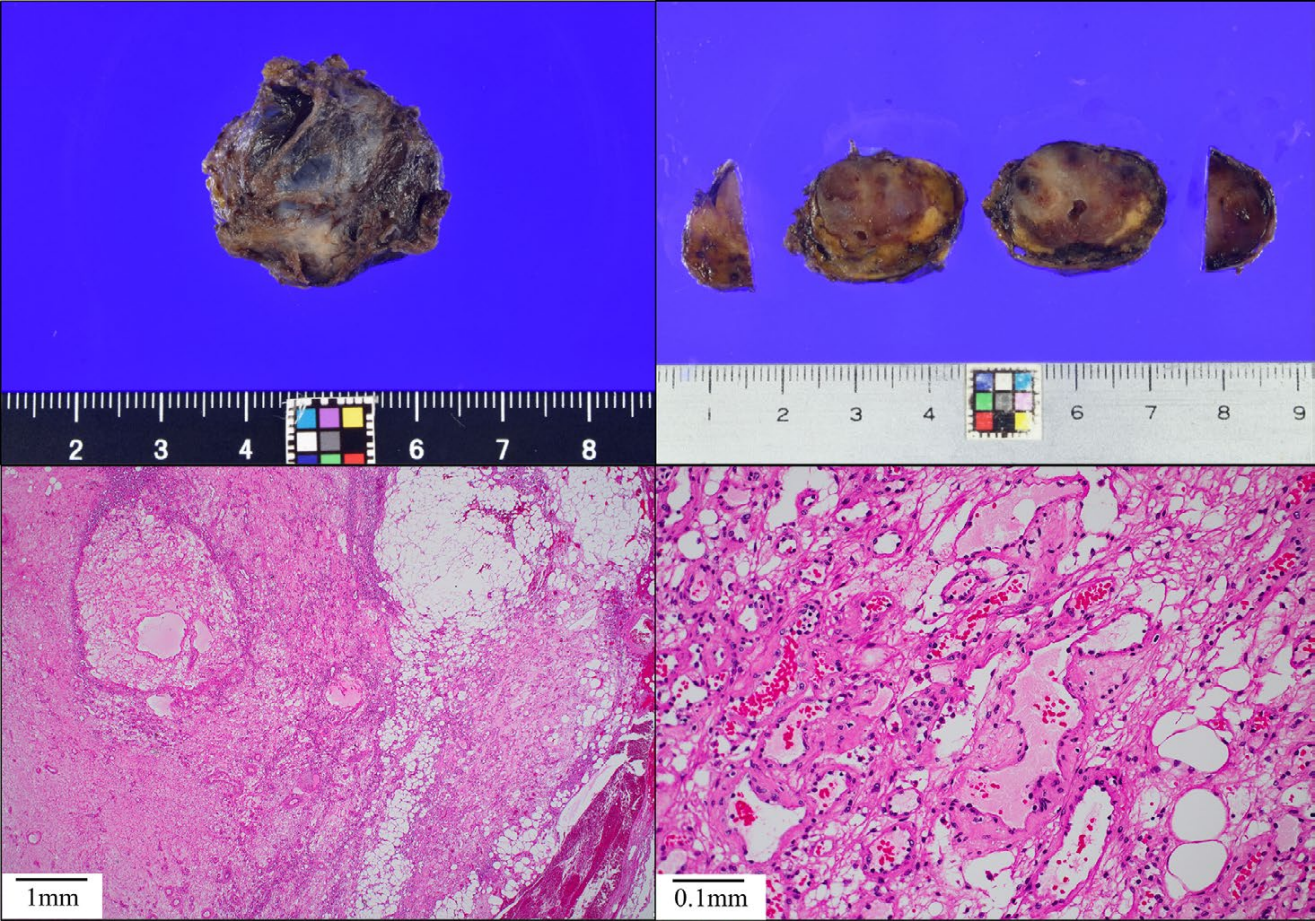
Surgical specimen and pathological findings. Surgical specimen was 28 × 26 × 18 mm tumor. The pathological findings showed the tumor consisted mainly of proliferating small blood vessels, suggesting cavernous hemangioma.

The patient was positioned in the lithotomy position, and the surgery was performed under general anesthesia. A 12‐mm port and a 5‐mm port were inserted into the umbilicus with a free access device (TOP Corp, Tokyo, Japan), and the other four ports were inserted. After docking the arms and instruments, the cockpit surgeon operated two forceps and a camera, while the assistant provided bedside support, including retraction of the surrounding tissue, as shown in Figures 2 and 3. We elevated the transverse mesocolon using sutures and identified the ligament of Treitz, jejunum, and inferior mesenteric vein (IMV). The tumor in the retroperitoneal region was identified from the dorsal side of the transverse colon mesentery, and an incision for the mesentery was performed to visualize the tumor. The tumor was able to be moved freely and to be dissected from the surrounding tissue with monopolar scissors or an ultrasonically activated device. The left renal vein was identified dorsal to the tumor, and the tumor was successfully resected without intraoperative complications. The total duration of surgery was 185 min and there was minimal blood loss. The patient was discharged without complications on postoperative day 6. The resected specimen was a 28 × 26 × 18 mm tumor surrounded by fibroadipose tissue (Figure 4). Histopathological findings revealed that the tumor consisted mainly of proliferating small blood vessels, and the tumor cells showed no atypia and no mitotic figures. These findings were consistent with cavernous hemangioma originating from the retroperitoneal area. The patient has remained well for more than 6 months following surgery.

## 3 Discussion

We reported the first case of a retroperitoneal cavernous hemangioma removed safely with the assistance of the SDLS. Retroperitoneal cavernous hemangioma is a rare type of lesion, accounting for approximately 1%–3% of retroperitoneal tumors [2]. According to the International Society for the Study of Vascular Anomalies (ISSVA) classification, vascular anomaly was broadly classified into two categories: vascular tumors involving proliferative changes in vascular endothelial cells and vascular malformations primarily consisting of structural vascular abnormalities [8]. Cavernous hemangioma has been defined as a venous malformation within the category of vascular malformations. In this report, despite extensive imaging including a PET‐CT, MIBG scintigraphy and somatostatin receptor scintigraphy, and performing EUS‐FNA biopsy during the preoperative evaluation, the diagnosis could not be confirmed. However, retrospective review of imaging studies revealed that the gradually enhancing tumor on contrast‐enhanced CT was consistent with a cavernous hemangioma. In the retroperitoneal region, tumors exhibiting such contrast enhancement patterns should be considered as a potential hemangioma.

In recent years, surgical assistance robots, such as the da Vinci surgical system (Intuitive Surgical Inc., Sunnyvale, CA, USA), hinotori surgical system (Medicaroid Inc., Kobe, Japan), or the Hugo RAS system (Medtronic, Minneapolis, MN, USA), have been widely introduced. The SDLS has unique features: eye tracking control, haptic feedback, and reusable instruments [4, 5]. The primary advantage of SDLS was the availability of insurance coverage in Japan. Moreover, the benefit of SDLS in this case was the ability to manipulate the forceps with a stable field of view unaffected by hand tremors. Haptic feedback from the forceps also enabled tissue resection without excessive force, thereby preventing damage to the tumor and surrounding tissues. These characteristics of SDLS were considered to have contributed to the safe execution of the surgery and effective resection. Conversely, robotic‐assisted surgery broadly faces the issue of high surgical costs, including high disposable costs. However, most of the forceps used with the SDLS are reusable, which is expected to reduce long‐term costs.

SDLS procedures have a disadvantage, namely, that operation times are longer than those of laparoscopic surgery. Factors considered to contribute to this drawback include the time required to change forceps compared to laparoscopic surgery, as well as the time needed to dock each arm to the robot. In this case, the docking time was 8 min which was considered slightly longer than in previous cases [9, 10]. This was due to the tumor being located in the deep retroperitoneal space, necessitating consideration of the surgical approach and the optimal placement of ports during the procedure. The surgical team deemed this additional time necessary and acceptable to safely perform the surgery and completely remove the tumor. Furthermore, unlike other surgical assistance robots, SDLS lacks articulation, and assistants' supports may occasionally be required to develop and maintain the surgical field. During the surgery described in this report as well, the support of an assistant was required. Specifically, when developing the surgical field, the assistant not only elevated the transverse mesocolon and tissues including IMV with strings, but also assisted by retracting the jejunum caudally to improve visualization of the surgical field. These actions were believed to have contributed to the stability of the surgical field, enabling safe resection. The additional factor contributing to the safe resection in this case was that the tumor was relatively small and located in the fixed area of the retroperitoneum. When tumors are larger or require extensive dissection, resection using the SDLS procedure may become difficult, and careful consideration of SDLS indications is warranted.

This is the first report of robot‐assisted resection with SDLS for retroperitoneal cavernous hemangioma. Since SDLS surgery offers significant benefits for both patients and surgeons, and may play a prominent role in the future of robot‐assisted surgery, further accumulation of cases is necessary.

## Author Contributions

Yoshiki Murase, Masayasu Aikawa, Yuichiro Watanabe, Taku Honma, Takuya Oba, Yumiko Kageyama, Kenichiro Takase, Yukihiro Watanabe, Hiroaki Ono, Katsuya Okada, Yasumitsu Hirano, Kojun Okamoto, and Isamu Koyama participated in the treatment of this patient and reviewed the manuscript. All authors read and approved the final version of the manuscript.

## Funding

The authors have nothing to report.

## Ethics Statement

This work does not require ethical considerations or approval. Informed consent to participate in this study was obtained from the patient.

## Consent

Written informed consent was obtained from the patient for publication of this case report and any accompanying images.

## Conflicts of Interest

The authors declare no conflicts of interest.

## Data Availability

Data sharing not applicable to this article as no datasets were generated or analysed during the current study.

## References

[1] F. Czeyda‐Pommersheim , C. Menias , A. Boustani , and M. Revzin , “Diagnostic Approach to Primary Retroperitoneal Pathologies: What the Radiologist Needs to Know,” Abdominal Radiology (New York) 46 (2021): 1062–1081. 32944824 10.1007/s00261-020-02752-8

[2] J. W. Braasch and A. B. Mon , “Primary Retroperitoneal Tumors,” Surgical Clinics of North America 47 (1967): 663–678. 6022980 10.1016/s0039-6109(16)38243-3

[3] L. Improta , D. Tzanis , T. Bouhadiba , K. Abdelhafidh , and S. Bonvalot , “Overview of Primary Adult Retroperitoneal Tumours,” European Journal of Surgical Oncology 46 (2020): 1573–1579. 32600897 10.1016/j.ejso.2020.04.054

[4] P. P. Rao , “Robotic Surgery: New Robots and Finally Some Real Competition!,” World Journal of Urology 36 (2018): 537–541. 29427003 10.1007/s00345-018-2213-y

[5] D. Stephan , H. Salzer , and F. Willeke , “First Experiences With the New Senhance® Telerobotic System in Visceral Surgery,” Visceral Medicine 34 (2018): 31–36. 29594167 10.1159/000486111 PMC5869585

[6] T. McKechnie , J. Khamar , R. Daniel , et al., “The Senhance Surgical System in Colorectal Surgery: A Systematic Review,” Journal of Robotic Surgery 17 (2023): 325–334. 36127508 10.1007/s11701-022-01455-0

[7] N. Melling , J. Barr , R. Schmitz , et al., “Robotic Cholecystectomy: First Experience With the New Senhance Robotic System,” Journal of Robotic Surgery 13 (2019): 495–500. 30264180 10.1007/s11701-018-0877-3

[8] K. Kunimoto , Y. Yamamoto , and M. Jinnin , “ISSVA Classification of Vascular Anomalies and Molecular Biology,” International Journal of Molecular Sciences 23 (2022): 2358. 35216474 10.3390/ijms23042358 PMC8876303

[9] T. Kulis , N. E. Samalavicius , T. Hudolin , et al., “Robotic‐Assisted Radical Prostatectomy: A Multicenter Experience With the Senhance Surgical System,” World Journal of Urology 42 (2024): 39. 38244127 10.1007/s00345-023-04732-1

[10] T. Sasaki , F. Tomohisa , M. Nishimura , et al., “Initial 30 Cholecystectomy Procedures Performed With the Senhance Digital Laparoscopy System,” Asian Journal of Endoscopic Surgery 16 (2023): 225–232. 36418001 10.1111/ases.13143

